# Development of a sensitive monoclonal antibody-based sandwich ELISA to detect Vip3Aa in genetically modified crops

**DOI:** 10.1007/s10529-020-02854-9

**Published:** 2020-03-05

**Authors:** Weixiao Liu, Xuri Liu, Chao Liu, Zhe Zhang, Wujun Jin

**Affiliations:** 1 Biotechnology Research Institute, Chinese Academy of Agricultural Sciences, Beijing, 100081 China; 2 Department of Food and Biological Engineering, Handan Polytechnic College, Handan, 056001 China; 3 State Key Laboratory of Stem Cell and Reproductive Biology, Institute of Zoology, Chinese Academy of Science, Beijing, 100101 China

**Keywords:** Vip3Aa, Monoclonal antibody, ELISA, Genetically modified crops, Cotton, Maize

## Abstract

**Objectives:**

To develop a sensitive monoclonal antibody-based sandwich enzyme-linked immunosorbent assay (ELISA) to detect Vip3Aa in genetically modified (GM) crops and their products.

**Results:**

Vegetative insecticidal proteins (Vips) are secreted by *Bacillus thuringiensis *(*Bt*) and are known to be toxic to Lepidoptera species. Vip3Aa family proteins, Vip3Aa19 and Vip3Aa20, were successfully applied in GM crops to confer an effective and persistent insecticidal resistance. A sensitive monoclonal antibody-based sandwich ELISA was developed to detect Vip3Aa in GM crops and their products. Two monoclonal antibodies were raised against the overexpressed and purified His-Vip3Aa20, were purified from mouse ascites and characterized. A sandwich ELISA method was developed using the 2G3-1D7 monoclonal antibody for capture and the biotin-labeled 1F9-1F5 monoclonal antibody for detection of Vip3Aa20. The linear detection range of the method was found to be approximately 31.25–500 pg/ml, with a sensitivity of 10.24 pg/ml.

**Conclusions:**

The established ELISA was effective for detecting Vip3Aa family proteins other than Vip3Aa8, and was successfully applied in the detection of Vip3Aa20 and Vip3Aa19 expressed in transgenic maize and cotton.

## Introduction

Some insect pathogenic microbiology synthesizes a large number of insecticidal proteins. These insecticidal proteins form inclusion bodies (such as Cry and Cyt proteins) or are secreted into the cultural medium (such as Vip and Sip proteins). Cry proteins are widely used in agricultural pest control (Ashouri 2004; Clive 2007; de Maagd et al. 2003; Estruch et al. 1997; Shelton 2012; Tabashnik et al. 2015). However, some important pests, such as *Agrotis ipsilon* and *Diabrotica *spp., exhibit high tolerance to Cry proteins and are seriously harmful to crops (Chakroun et al. 2016; Chattopadhyay and Banerjee 2018). In 1996, Vip proteins were screened from the culture supernatant of *Bacillus thuringiensis* (Estruch et al. 1996) and found to be toxic to a wide range of Lepidoptera species, some of which show tolerance or low susceptibility to Cry proteins.

Vip proteins are divided into four families based on the homology of their amino acid sequences: Vip1, Vip2, Vip3 and Vip4 (https://www.btnomenclature.info/). Among them, the Vip3Aa protein has been found to be toxic to most Lepidoptera insects. Notably, Vip3Aa is highly toxic to *Agrotis*, which is resistant to Cry. Vip3Aa has also been applied against species of *Spodoptera*, which are insensitive to Cry toxicity (Donovan et al. 2001; Liu et al. 2007; Selvapandiyan et al. 2001). Furthermore, *vip3Aa* genes have been successfully transferred into cotton and maize, as patented in the United States in 2009 (Adamczyk and Mahaffey 2008; Kurtz et al. 2007).

The rapid development of genetically modified (GM) crops has resulted in growing public concern that GM crops may lead to unexpected food and environmental safety issues. Thus, accurate detection of foreign genes and their products in GM crops is becoming increasingly important and urgent. There are numerous methods for detecting foreign genes in transgenic crops (Kamle et al. 2017; Salisu et al. 2017), and the most direct detection method is based on the gene-encoded protein. ELISA is a specific, sensitive, and convenient method for protein detection. Furthermore, the method is precise, reproducible and employs stable reagents and inexpensive equipment. Therefore, ELISA is applicable for routinely detecting foreign gene-encoded proteins in GM crops and their products (Albright et al. 2016a, 2016b; Kamle et al. 2011b, 2013).

Based on this information, we used overexpressed His-Vip3Aa20 as an immunogen to generate mouse monoclonal antibodies (mAbs) that recognize certain surface components of the protein. These antibodies were then employed to develop a sandwich ELISA for sensitive, direct and convenient measurement of the Vip3Aa concentration in GM crops and their products. To the best of our knowledge, this is the first quantitative monoclonal antibody-based ELISA method reported for the sensitive detection of Vip3Aa proteins.

## Materials and methods

### Reagents, strains and animals

DNA markers, *pfu* polymerase and *Escherichia coli* (*Trans* 10 and BL21) chemically competent cells were obtained from TransGen Biotech. Ni-Resin, Superdex 200 and Protein A-Sepharose columns were purchased from GE Healthcare. A protein marker was purchased from Fermentas. HyClone DMEM and fetal calf serum (FCS) were purchased from Thermo. All reagents were of analytical grade. Complete and incomplete Freund’s adjuvant, 50% polyethylene glycol (PEG), hypoxanthine/aminopterin/thymidine (HAT) and hypoxanthine/thymidine (HT) were obtained from Sigma-Aldrich and proteinase inhibitor cocktails from Roche. Goat anti-mouse immuno-globulin horseradish peroxidase conjugate was obtained from Univ-bio (Shanghai, China) and avidin-horseradish peroxidase conjugate from Invitrogen. BABL/c mice were obtained from SBF company (Beijing, China).

### His-Vip3Aa20 protein expression

The *vip3Aa20* gene was amplified from GM maize MIR162 genomic DNA and subcloned into the pET28a plasmid. pET28a-*vip3Aa1/19* and pET28a-*vip3Aa7/10* were generated by site-directed mutagenesis(Palma et al. 2017). The *vip3Aa14* and *vip3Aa8* genes were chemically synthesized and subcloned into the pET28a plasmid. The reconstituted plasmids were transformed into *E. coli* BL21 competent cells. Overexpressed recombinant His-Vip3Aa20, His-Vip3Aa1/19, His-Vip3Aa7/10, His-Vip3Aa14 and His-Vip3Aa8 proteins were purified with His-affinity purification (native or denature) and further fractionated by size-exclusion chromatography using a Superdex200 column with 50 mM Tris HCl, pH 7.6, 150 mM potassium chloride and 5% glycerol.

### Mice immunization protocols

The immunization protocol followed conventional subcutaneous (s.c.) injection, with slight modifications (Li et al. 2009). Eight-week-old male BALB/c mice were immunized with immunogen [His-Vip3Aa20 dissolved in 250 μ L of sterile phosphate-buffered saline (PBS) adjuvant with complete Freund's adjuvant or incomplete Freund's adjuvant] at the nape of the neck at weeks 1, 4, 6 and 8. The immunizing dose was fixed at 50 μg by subcutaneous multi-point injection per mouse each time. Three days prior to cell fusion, the mice were boosted with 50 μg of adjuvant-free immunogen. Blood samples were collected from the mouse tail, and the titers were determined by ELISA.

### Fusion, hybridoma screening and mAb generation

Seven days after the final booster immunization, a single-cell suspension was aseptically prepared in RPMI-1640 medium from mouse spleen samples. The cell suspensions were mixed with murine myeloma cells SP2/0 (Genecreate Biological Engineering Co.) at a ratio of 10:1. The mixed cell suspension was centrifuged, and the supernatant was completely removed. The combined splenocyte suspension and sp2/0 cells were fused using a modification of the method described by Galfre et al*.* (Galfre and Milstein 1981). One milliliter of 50% PEG1450 was added dropwise to the mixed cell pellet in a 50-ml centrifuge tube over 90 s. The mixture was gently stirred while PEG1450 was added, and the mixture was then allowed to incubate for 1 min. Fusion was stopped by dropwise addition of 50 ml of RPMI-1640 to the mixture. The temperature was maintained at 37 °C throughout the entire procedure. The cell suspension was then centrifuged at 1500 rpm for 5 min. The precipitated cells were gently resuspended in 20 ml of HAT complete liquid medium, and the fusion suspension was gently mixed with 80 ml of semi-solid complete medium containing 1 g of methyl cellulose. Finally, 1.5 ml of the fusion cell suspension was distributed into 6-well plates and incubated in 5% CO_2_ at 37 °C.

After the cells were cultured for 7–10 days, many white dots suggestive of monoclonal hybridomas had formed on the semi-solid selective complete medium. These white dots were transferred to 96-well culture plates to enable screening for hybridoma cell lines secreting the anti-Vip3Aa20 antibody. Next, the absorbance of the culture supernatant of anti-Vip3Aa20 antibody-secreting hybridoma cell lines was evaluated at 450 nm (A450). Anti-Vip3Aa20 antibody-secreting hybridoma cell lines with an A450 value higher than 2.0 were injected into the abdomens of 10-week-old BALB/c mice (Daginakatte et al. 1999; Dong et al. 2016; Esch et al. 2012; Narat et al. 2004), and the ascitic fluid was collected approximately seven days later. The mAbs were purified from the ascitic fluid using the saturated ammonium sulfate precipitation method and subsequently purified using protein A-Sepharose columns (Groopman et al. 1984).

### Antibody characterization

Antibody titers were determined via indirect ELISA. The antibody titer was defined as the highest antibody dilution that gave an absorbance greater than 2.1-fold of the background absorbance of PBS (negative control)(Martin et al. 2000). Subclass assessments of the mAbs were performed by direct ELISA using a commercially available kit from Sigma(Azimzadeh and Van Regenmortel 1991; Beatty et al. 1987).

### Protein sample preparation

MIR162 maize standard substance (AOCS 1208-A, USA) and negative maize samples and COT102 cotton standard substance (AOCS 1012-C, USA) and negative cotton samples were ground in liquid N_2_, resuspended in lysate buffer (100 mM Tris, pH 7.5, 300 mM NaCl, 5 mM EDTA, 5% glycerol, 0.1% SDS, protease cocktail and 1 mM PMSF) at 4 °C for 30 min and then centrifuged at 12,000 rpm and 4 °C for 15 min. The supernatants were collected and mixed with loading buffer for SDS-PAGE/Western blotting analysis or diluted to an appropriate concentration for ELISA.

### Western blotting analysis

The His-Vip3Aa20 protein, MIR162 maize standard substance and negative maize samples, and gradient concentrations of His-Vip3Aa20, His-Vip3Aa1/19, His-Vip3Aa7/10, His-Vip3Aa14 and His-Vip3Aa8 were electrophoresed on a 4–12% nuPAGE gel (Invitrogen, USA) and transferred onto polyvinylidene fluoride (PVDF) membranes (Millipore, US). The membranes were incubated with 5 μg/ml His-Vip3Aa20 mAbs at 4 °C overnight after nonspecific sites were blocked with 5% skim milk. The membranes were washed three times and incubated with Alexa Fluor™ 680 goat anti-mouse IgG (H+L) (Invitrogen, USA) for 1 h at room temperature. The membranes were processed using an Odyssey scanner (LI-COR, USA).

### Sandwich ELISA development

The wells of an ELISA plate were coated with 100 μ l of 2 μg/ml capture antibody and incubated overnight at 4 °C. Three washes with PBST were performed to remove unbound antibody. Each well was blocked with 200 μ l of 5% skim milk and incubated at 37 °C for 90 min. The washing step was repeated, and 100 μl of His-Vip3Aa20 protein serially diluted in PBS was added to each well. The plate was incubated for 2 h at 37 °C. Next, 1 μg/ml of biotin-labeled anti-His Vip3Aa20 mAb was added to each well and incubated for 1 h at 37 °C. The plates were washed and incubated with avidin-HRP for 30 min at 37 °C. The plate was then washed five times, and 90 μ l of TMB solution was added to each well. The reaction was terminated by the addition of 50 μl of 1 M H_2_SO_4_, and the absorbance at 450 nm was measured.

### Detection of Vip3Aa by the sandwich ELISA

One hundred microliters of Vip3Aa20 protein, Vip3Aa1/19, MIR162, COT12 or negative sample was added to each antibody-coated well. The plate was covered with an adhesive strip and incubated for 2 h at 37 °C. Next, the liquid in each well was removed, and 100 μl of biotin-labeled antibody was added. The wells were incubated for 1 h at 37 °C and then washed three times with PBST (Phosphate Buffer Solution Tween-20). After the last wash, any remaining liquid was removed by aspiration. Next, 100 μl of avidin-HRP was added to each well. The plate was covered with a new adhesive strip and incubated for 1 h at 37 °C, followed by five washing steps. Next, 90 μ l of TMB solution was added to each well, the reaction was terminated by the addition of 50 μl of 1 M H_2_SO_4_, and absorbance at 450 nm was measured.

### Statistical analysis

Data are expressed as the average value and standard deviation (SD). The limit of detection (LOD) was calculated using the standard formula, with a slight modification (Dixit et al. 2011; Vashist 2013; Vashist et al. 2014).

### Protein sequence alignment

Sequences of Vip3Aa family proteins were downloaded from https://www.btnomenclature.info/ and aligned using DNAMAN software.

## Results

### His-Vip3Aa20 protein expression and purification

**Fig. 1:**
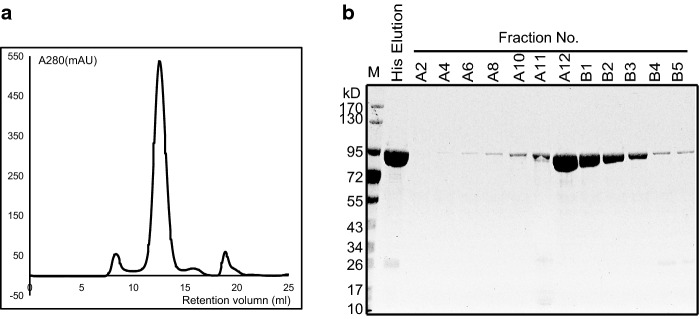
Purification of the overexpressed His-Vip3Aa20 protein. **a** Size exclusion chromatography analysis of His-Vip3Aa20. **b** SDS-PAGE analysis of His-Vip3Aa20 fractions from size-exclusion chromatography

The vip3Aa20 gene was amplified from GM MIR162 genomic DNA and subcloned into the pET28a expression vector for overexpression and purification. His-Vip3Aa20 was successfully expressed in E. coli BL21 cells. After nickel affinity purification, the protein was further fractionated by size-exclusion chromatography on a Superdex200 column (Fig. 1a). The molecular weight of the overexpressed His-Vip3Aa20 is approximately 90 kD (Fig. 1b). Purified His-Vip3Aa20 was used to immunize 8-week-old male BALB/c mice.

### Anti-His-Vip3Aa20 mAb preparation and characterization

**Fig. 2:**
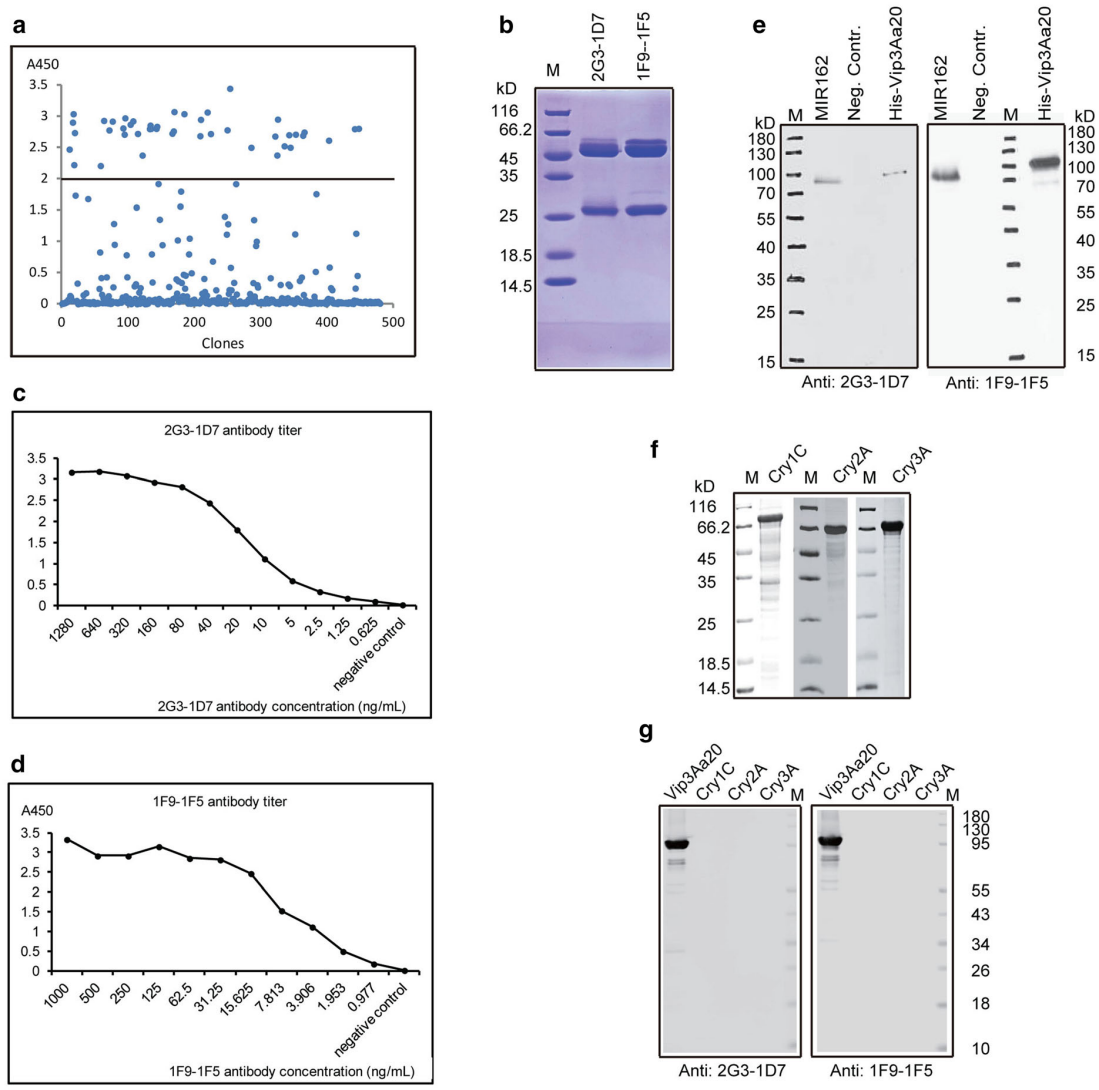
Characterization of anti-His-Vip3Aa20 mAbs. **a** The screening of hybridomas. **b** SDS-PAGE analysis of purified mAbs 1F9-1F5 and 2G3-1D7. **c** 2G3-1D7 titer. **d** 1F9-1F5 titer. **e** Western blotting analysis of Vip3Aa20 in GM maize MIR162 and His-Vip3Aa20 against 5 µg/ml mAbs 1F9-1F5 and 2G3-1D7. **f** SDS-PAGE analysis of Cry1C, Cry2A and Cry3A, proteins to which insects show resistance. **g** Western blotting analysis of His-Vip3Aa20, Cry1C, Cry2A and Cry3A against 5 µg/ml mAbs 1F9-1F5 and 2G3-1D7

Anti-His-Vip3Aa20 mAbs were prepared according to antibody preparation techniques. More than 450 hybridomas were screened using indirect ELISA. Hybridomas with A450 values greater than 2.0 were selected for further subcloning (Fig. 2a). Finally, two anti-His-Vip3Aa20 antibody-secreting hybridoma clones (named 1F9-1F5 and 2G3-1D7) were screened, expanded and injected into the abdomens of 10-week-old BALB/c mice for ascitic fluid preparation. The mAbs 1F9-1F5 and 2G3-1D7 were purified from ascitic fluid using saturated ammonium sulfate precipitation and a protein A-Sepharose column. SDS-PAGE results demonstrated that the purified antibodies 1F9-1F5 and 2G3-1D7 are 55-kDa (heavy chain) and 25-kDa (light chain), as expected, and that extraneous proteins were eliminated (Fig. 2b). The titers of the purified mAbs 1F9-15 and 2G3-1D7 were 1:4,900,000 and 1:8,600,000, respectively (Fig. 2c, d). The isotypes of the two mAbs were determined to be IgG1 and IgG2a (Table 1). His-Vip3Aa20 and Vip3Aa20 in MIR162 standard substance were successfully recognized by the mAbs (Fig. 2e). Several proteins to which insects exhibit resistance, such as Cry1C, Cry2A and Cry3A, which have been widely and successfully applied in transgenic crops, were used to test the cross-reactivity of the 2G3-1D7 and 1F9-1F5 mAbs (Fig. 2f, g). These mAbs specifically recognized the Vip3Aa20 protein but not the other proteins assessed. These 2G3-1D7 and 1F9-1F5 mAbs were used for subsequent analyses.

**Table 1:** Anti-His-Vip3Aa20 mAbs selected for ELISA

Item	Antigen	Property	Host	Antibody subtype	Titers	Application
1F9-1F5	His-Vip3Aa20	Monoclonal	Mouse	IgG1	1:4,900,000	ELISA, WB
2G3-1D7	His-Vip3Aa20	Monoclonal	Mouse	IgG2a	1:8,600,000	ELISA, WB

### Optimization of the sandwich ELISA and standard curves

**Fig. 3:**
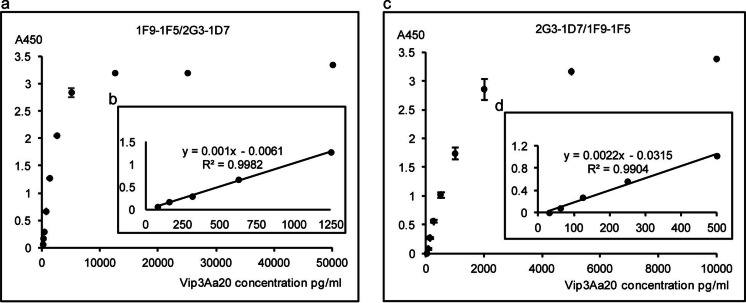
Double-antibody sandwich ELISA for the detection of Vip3Aa20. **a** Standard curve of the sandwich ELISA with the mAb 1F9-1F5 as the capture antibody and biotin-labeled mAb 2G3-1D7 as the detection antibody. **b** The inset shows the linear detection from the standard curve in (**a)**. **c** Standard curve of the sandwich ELISA with the mAb 2G3-1D7 as the capture antibody and the biotin-labeled mAb 1F9-1F5 as the detection antibody. **d** The inset shows the linear detection from the standard curve in **c**

The mAb 1F9-1F5 served as the capture antibody, and the biotin-labeled mAb 2G3-1D7 served as the detection antibody. Standard dilutions were obtained by serial dilution with an initial His-Vip3Aa20 protein concentration of 50 ng/ml (Fig. 3a) and used to construct a standard curve, with the equation y = 0.001x–0.0061. The working range of the assay was defined as the part of the curve with a linear coefficient of R^2^ > 0.99. The linear range included concentrations of 78.125 pg/ml to 1.25 ng/ml, with an LOD of 17.148 pg/ml.

2G3-1D7 was also used as the capture antibody, with biotin-labeled mAb 1F9-1F5 as the detection antibody. Standard dilutions of an initial His-Vip3Aa20 protein concentration of 10 ng/ml (Fig. 3b) were used to construct a standard curve, with y = 0.0022x–0.0315. The working range of the assay was defined as above, and the linear range included concentrations of 31.25 pg/ml to 500 pg/ml, with an LOD of 10.242 pg/ml. Due to its higher sensitivity, this second ELISA method was selected for further analysis. The variability of this ELISA method was examined using the coefficient of variation (CV). As shown in Table 2, the intra- and interassay variation values of this ELISA were 5.972% and 4.661%, respectively. These data indicate that the sandwich ELISA developed for Vip3Aa20 detection is a convenient and sensitive assay.

**Table 2:** Intra- and interassay coefficient of variation for the ELISA system

Theoretical value (pg/ml)	Average value (pg/ml)	Standard deviation (SD)	Coefficient of variation (CV %)	Average CV (%)
Intra-assay coefficient of variation (n = 8)				
1000	1031.116	0.128	6.589	5.972
250	276.968	0.046	5.812	
62.5	57.108	0.014	5.515	
Interassay coefficient of variation (n = 24)				
1000	1029.059	0.131	0.733	4.661
250	275.684	0.051	6.496	
62.5	51.679	0.016	6.755	

### Detection of Vip3Aa family proteins by the two screened mAbs and established ELISA method

**Fig. 4:**
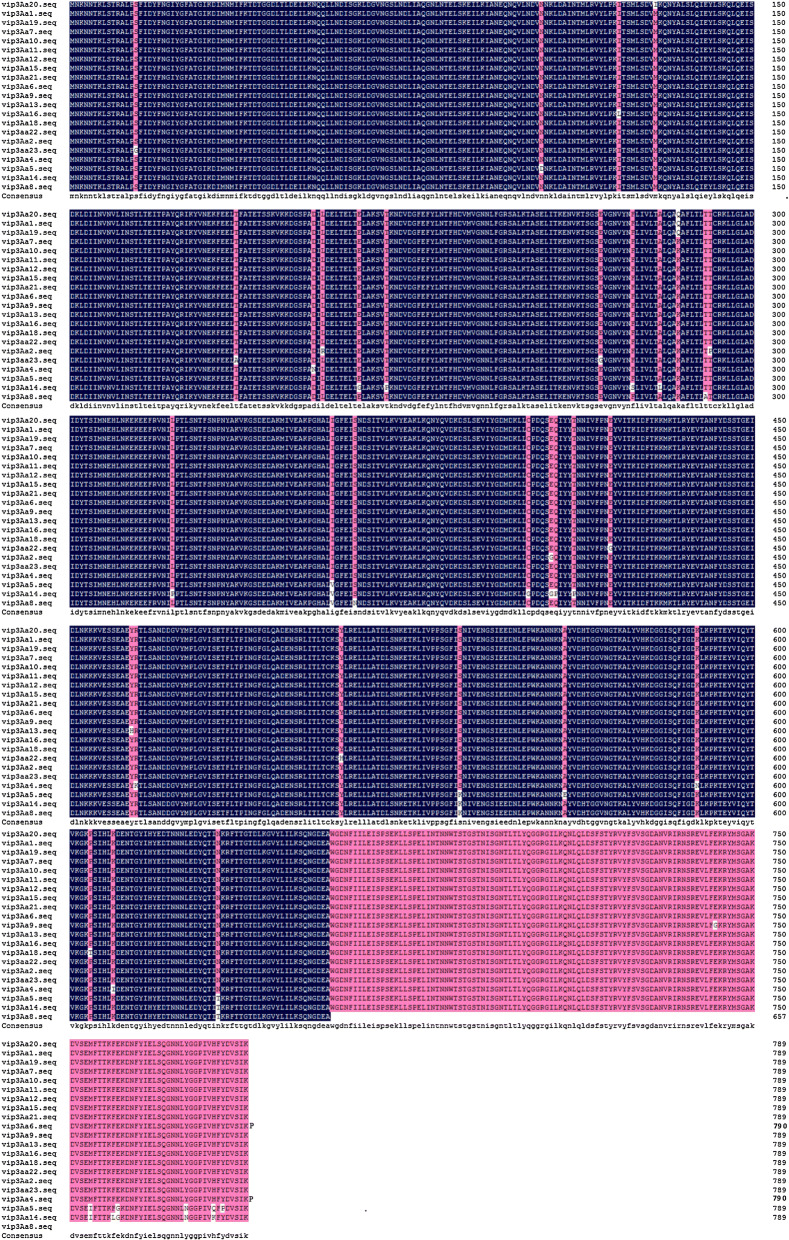
Multiple-sequence alignment of Vip3Aa proteins. Sequence identity is indicated by shading. All sequences were downloaded from https://www.btnomenclature.info/

**Fig. 5:**
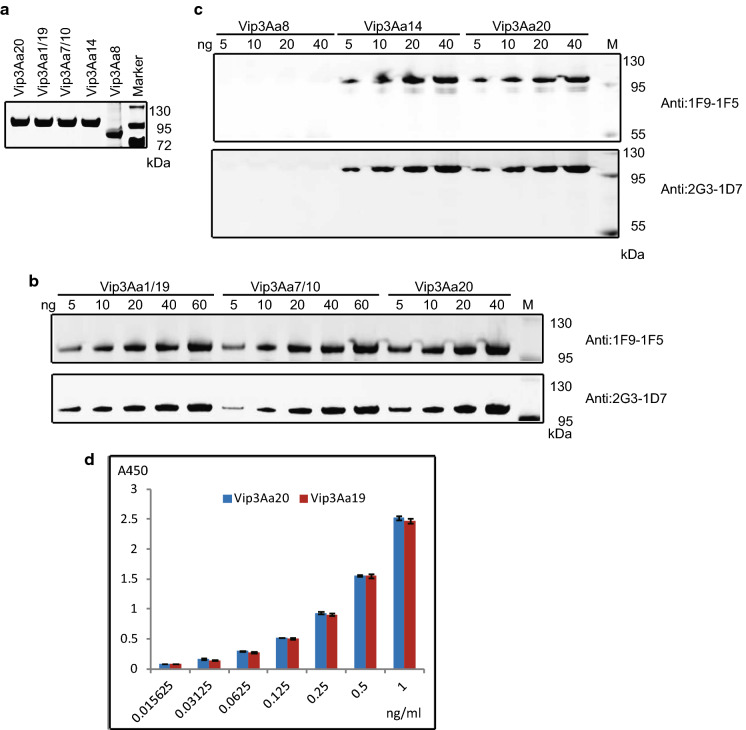
Vip3Aa family proteins detected by the mAbs screened and ELISA method developed. **a** SDS-PAGE analysis of purified Vip3Aa family proteins. **b** Western blotting analysis of Vip3Aa1/19 and Vip3Aa7/10 against 5 µg/ml mAbs 1F9-1F5 and 2G3-1D7. **c** Western blotting analysis of Vip3Aa14 and Vip3Aa8 against 5 µg/ml mAbs 1F9-1F5 and 2G3-1D7. **d** ELISA analysis of a gradient dilution of Vip3Aa19

The sequences of Vip3Aa family proteins were downloaded from https://www.btnomenclature.info/ and analyzed using DNAMAN (Fig. 4). Two proteins with the highest (Vip3Aa1/19 and Vip3Aa7/10) and lowest (Vip3Aa14 and Vip3Aa8) sequence consistency were expressed and purified. With the exception of His-Vip3Aa8, which has a molecular weight of approximately 75 kDa, the molecular weights of the other proteins were identical to that of His-Vip3Aa20, which is 90 kDa (Fig. 5a). His- Vip3Aa1/19, His-Vip3Aa7/10 and His- Vip3Aa14 were successfully identified by the purified mAbs 1F9-15 and 2G3-1D7, whereas was not His-Vip3Aa8 (Fig. 5b, c). Because Vip3Aa19 and Vip3Aa20 have been applied in transgenic crops, His-Vip3Aa19 and His-Vip3Aa20 were diluted in a concentration gradient and detected by the ELISA (Fig. 5d). The results showed the ELISA method to also be suitable for detecting Vip3Aa family proteins other than Vip3Aa8.

### Detection of Vip3Aa in GM maize MIR162 and cotton COT102 samples

Protein samples were prepared from GM maize MIR162 and cotton COT12 standard substances and then diluted and detected as described in the Materials and Methods section. The Vip3Aa20 content in GM maize MIR162 (x = 27.73 ± 0.27 μg/g) and the Vip3Aa19 content in GM cotton COT12 (x = 75.96 ± 0.73 ng/g) were calculated using the equation shown in Fig. 3b.

## Discussion

Vip3Aa proteins secreted by *Bacillus thuringiensis* have been screened for Lepidoptera toxicity and applied in GM crops (such as maize and cotton). However, with the rapid development of GM crops, there is heightened concern that these crops may lead to unexpected food safety and environmental safety issues. Thus, sensitive detection of exogenous proteins has become increasingly important. Numerous methods for detecting and monitoring transgenic crops have been established. The most popular techniques are polymerase chain reaction (PCR) and ELISA based on foreign DNA and protein, respectively. Due to advantages of sensitive and high throughput, PCR has been widely used in many GM crop detection (Aguilera et al. 2009; Kamle et al. 2011a). Nevertheless, proteins not only have functions but also act as toxins or allergens. As monitoring, tracing and quantifying foreign proteins in GM crops are crucial, ELISA detection methods have been promoted. The Cry protein is among first and most widely used in agricultural application. Indeed, several ELISA methods for monitoring Cry1Ab, Cry1Ac and Cry1Ie were established (Walschus et al.^2002^; Wang et al. 2007; Zhang et al. 2016). In this study, we describe a sensitive monoclonal antibody-based ELISA method for the detection of Vip3Aa in GM crops and their products.

Compared with the previously developed triple antibody sandwich ELISA for Vip3A (Kumar 2012), the ELISA method developed in the present study is more sensitive. Furthermore, we clearly indicate the scope of the application of this ELISA method, which is suitable for detecting Vip3Aa family proteins other than Vip3Aa8. Specifically, our results indicate that the *C*-terminal sequences of Vip3Aa family proteins are recognized by the two mAbs screened, 1F9-1F5 and 2G3-1D7 (Fig. 5b, c). The newly developed ELISA method is a sensitive technique for determining the Vip3Aa content in GM crops and their products.

## Conclusions

This report describes a sensitive monoclonal antibody-based ELISA for the detection of Vip3Aa in GM crops. The titers of the screened mAbs 2G3-1D7 and 1F9-1F5 were 1:8,600,000 and 1:4,900,000, respectively. This sensitive ELISA method was developed to detect Vip3Aa family proteins (other than Vip3Aa8), with a working range of 31.25–500 pg/ml and an LOD of 10.242 pg/ml. The ELISA method performed well in recovery tests and can be used for quantitative and convenient detection of Vip3Aa proteins in maize and cotton samples.

## References

[CR1] Adamczyk JJ, Mahaffey JS (2008). Efficacy of Vip3a and Cry1ab transgenic traits in cotton against various lepidopteran pests. Fla Entomol.

[CR2] Aguilera M, Querci M, Pastor S, Bellocchi G, Milcamps A, Eede G (2009). Assessing copy number of MON 810 integrations in commercial seed maize varieties by 5' event-specific real-time PCR validated method coupled to 2(-Delta Delta CT). Anal Food Anal Method.

[CR3] Albright VC, Hellmich RL, Coats JR (2016). Enzyme-linked immunosorbent assay detection and bioactivity of Cry1ab protein fragments. Environ Toxicol Chem.

[CR4] Albright VC, Hellmich RL, Coats JR (2016). A review of Cry protein detection with enzyme-linked immunosorbent assays. J Agr Food Chem.

[CR5] Ashouri A (2004). Transgenic-Bt potato plant resistance to the colorado potato beetle affect the aphid parasitoid Aphidius nigripes. Commun Agric Appl Biol Sci.

[CR6] Azimzadeh A, Van Regenmortel MH (1991). Measurement of affinity of viral monoclonal antibodies by ELISA titration of free antibody in equilibrium mixtures. J Immunol Methods.

[CR7] Beatty JD, Beatty BG, Vlahos WG (1987). Measurement of monoclonal antibody affinity by non-competitive enzyme immunoassay. J Immunol Methods.

[CR8] Chakroun M, Banyuls N, Bel Y, Escriche B, Ferre J (2016). Bacterial vegetative insecticidal proteins (Vip) from entomopathogenic bacteria. Microbiol Mol Biol Rev.

[CR9] Chattopadhyay P, Banerjee G (2018). Recent advancement on chemical arsenal of Bt toxin and its application in pest management system in agricultural field. Biotechnology.

[CR10] Clive J (2007). The global status of the commercialized biotechnological/genetically modified crops: 2006. Tsitol Genet.

[CR11] Daginakatte GC, Chard-Bergstrom C, Andrews GA, Kapil S (1999). Production, characterization, and uses of monoclonal antibodies against recombinant nucleoprotein of elk coronavirus. Clin Diagn Lab Immun.

[CR12] de Maagd RA, Bravo A, Berry C, Crickmore N, Schnepf HE (2003). Structure, diversity, and evolution of protein toxins from spore-forming entomopathogenic bacteria. Annu Rev Genet.

[CR13] Dixit CK, Vashist SK, MacCraith BD, O'Kennedy R (2011). Multisubstrate-compatible ELISA procedures for rapid and high-sensitivity immunoassays. Nat Protoc.

[CR14] Dong S (2016). Production and characterization of monoclonal antibody broadly recognizing Cry1 toxins by use of designed polypeptide as Hapte. Anal Chem.

[CR15] Donovan WP, Donovan JC, Engleman JT (2001). Gene knockout demonstrates that vip3A contributes to the pathogenesis of Bacillus thuringiensis toward Agrotis ipsilon and Spodoptera exigua. J Invertebr Pathol.

[CR16] Esch AM, Thompson NE, Lamberski JA, Mertz JE, Burgess RR (2012). Production and characterization of monoclonal antibodies to estrogen-related receptor alpha (ERR alpha) and use in immunoaffinity chromatography. Protein Expres Purif.

[CR17] Estruch JJ, Carozzi NB, Desai N, Duck NB, Warren GW, Koziel MG (1997). Transgenic plants: An emerging approach to pest control. Nat Biotechnol.

[CR18] Estruch JJ, Warren GW, Mullins MA, Nye GJ, Craig JA, Koziel MG (1996). Vip3A, a novel Bacillus thuringiensis vegetative insecticidal protein with a wide spectrum of activities against lepidopteran insects. Proc Natl Acad Sci USA.

[CR19] Galfre G, Milstein C (1981). Preparation of monoclonal antibodies: strategies and procedures. Methods Enzymol.

[CR20] Groopman JD, Trudel LJ, Donahue PR, Marshak-Rothstein A, Wogan GN (1984). High-affinity monoclonal antibodies for aflatoxins and their application to solid-phase immunoassays. Proc Natl Acad Sci USA.

[CR21] Kamle M, Kumar P, Patra JK, Bajpai VK (2017). Current perspectives on genetically modified crops and detection methods. Biotechnology.

[CR22] Kamle S, Kumar A, Bhatnagar RK (2011). Development of multiplex and construct specific PCR assay for detection of cry2Ab transgene in genetically modified crops and product. GM crops.

[CR23] Kamle S, Ojha A, Kumar A (2011). Development of an enzyme linked immunosorbant assay for the detection of Cry2Ab Protein in transgenic plants. GM crops.

[CR24] Kamle S, Ojha A, Kumar A (2013). Development of enzyme-linked immunosorbent assay for the detection of Bt protein in transgenic cotton. Methods Mol Biol.

[CR25] Kumar R (2012). Development of ELISA for the detection of transgenic vegetative insecticidal protein in GM crops/produce.. Food Addit Contam Part A.

[CR26] Kurtz RW, McCaffery A, O'Reilly D (2007). Insect resistance management for Syngenta's VipCot (TM) transgenic cotton. J Invertebr Pathol.

[CR27] Li PW (2009). Development of a class-specific monoclonal antibody-based ELISA for aflatoxins in peanut. Food Chem.

[CR28] Liu J, Song F, Zhang J, Liu R, He K, Tan J, Huang D (2007). Identification of vip3A-type genes from Bacillus thuringiensis strains and characterization of a novel vip3A-type gene. Lett Appl Microbiol.

[CR29] Martin DA, Muth DA, Brown T, Johnson AJ, Karabatsos N, Roehrig JT (2000). Standardization of immunoglobulin M capture enzyme-linked immunosorbent assays for routine diagnosis of arboviral infections. J Clin Microbiol.

[CR30] Narat M, Bicek A, Vadnjal R, Bencina D (2004). Production, characterization and use of monoclonal antibodies recognizing IgY epitopes shared by chicken, turkey, pheasant, peafowl and sparrow. Food Technol Biotech.

[CR31] Palma L (2017). The Vip3Ag4 insecticidal protoxin from bacillus thuringiensis adopts a tetrameric configuration that is maintained on proteolysis. Toxins.

[CR32] Salisu IB, Shahid AA, Yaqoob A, Ali Q, Bajwa KS, Rao AQ, Husnain T (2017). Molecular approaches for high throughput detection and quantification of genetically modified crops: a review front. Plant Sci.

[CR33] Selvapandiyan A, Arora N, Rajagopal R, Jalali SK, Venkatesan T, Singh SP, Bhatnagar RK (2001). Toxicity analysis of *N*- and *C*-terminus-deleted vegetative insecticidal protein from Bacillus thuringiensis. Appl Environ Microbiol.

[CR34] Shelton AM (2012). Genetically engineered vegetables expressing proteins from Bacillus thuringiensis for insect resistance: successes, disappointments, challenges and ways to move forward. GM Crops Food.

[CR35] Tabashnik BE (2015). Dual mode of action of Bt proteins: protoxin efficacy against resistant insects. Sci Rep.

[CR36] Vashist SK (2013). A sub-picogram sensitive rapid chemiluminescent immunoassay for the detection of human fetuin A. Biosens Bioelectron.

[CR37] Vashist SK, Marion Schneider E, Lam E, Hrapovic S, Luong JH (2014). One-step antibody immobilization-based rapid and highly-sensitive sandwich ELISA procedure for potential in vitro diagnostics. Sci Rep.

[CR38] Walschus U, Witt S, Wittmann C (2002). Development of monoclonal antibodies against Cry1Ab protein from Bacillus thuringiensis and their application in an ELISA for detection of transgenic Bt-maize. Food Agr Immunol.

[CR39] Wang S, Guo AY, Zheng WJ, Zhang Y, Qiao H, Kennedy IR (2007). Development of ELISA for the determination of transgenic Bt-cottons using antibodies against Cry1Ac protein from Bacillus thuringiensis HD-73. Eng Life Sci.

[CR40] Zhang YW, Zhang W, Liu Y, Wang JH, Wang GY, Liu YJ (2016). Development of monoclonal antibody-based sensitive ELISA for the determination of Cry1Ie protein in transgenic plant. Anal Bioanal Chem.

